# Male manipulation impinges on social-dependent tumor suppression in *Drosophila melanogaster* females

**DOI:** 10.1038/s41598-024-57003-3

**Published:** 2024-03-17

**Authors:** Perla Akiki, Pierre Delamotte, Mickael Poidevin, Erwin L. van Dijk, Apolline J. R. Petit, Arnaud Le Rouzic, Frederic Mery, Frederic Marion-Poll, Jacques Montagne

**Affiliations:** 1 Institut for Integrative Biology of the Cell (I2BC), UMR 9198, CNRS, CEA, Université Paris-Saclay, 91190 Gif-sur-Yvette, France; 2 UMR EGCE, CNRS, Université Paris-Saclay, 91190 Gif-sur-Yvette, IRD France; 3 Laboratoire Biométrie Et Biologie Evolutive, UMR 5558, CNRS, Université Claude Bernard Lyon 1, 69622 Villeurbanne Cedex, France; 4 Université Paris-Saclay, AgroParisTech, 91123 Palaiseau Cedex, France

**Keywords:** Nervous system, Brain, Behavior, Virginity, Fertilization, Sex-peptide, Cancer models, Behavioural genetics, Cancer, Evolution, Genetics, Physiology, Environmental social sciences

## Abstract

Physiological status can influence social behavior, which in turn can affect physiology and health. Previously, we reported that tumor growth in *Drosophila* virgin females depends on the social context, but did not investigate the underlying physiological mechanisms. Here, we sought to characterize the signal perceived between tumorous flies, ultimately discovering that the tumor suppressive effect varies depending on reproductive status. Firstly, we show that the tumor suppressive effect is neither dependent on remnant pheromone-like products nor on the microbiota. Transcriptome analysis of the heads of these tumorous flies reveals social-dependent gene-expression changes related to nervous-system activity, suggesting that a cognitive-like relay might mediate the tumor suppressive effect. The transcriptome also reveals changes in the expression of genes related to mating behavior. Surprisingly, we observed that this social-dependent tumor-suppressive effect is lost in fertilized females. After mating, *Drosophila* females change their behavior—favoring offspring survival—in response to peptides transferred via the male ejaculate, a phenomenon called “male manipulation”. Remarkably, the social-dependent tumor suppressive effect is restored in females mated by sex-peptide deficient males. Since male manipulation has likely been selected to favor male gene transmission, our findings indicate that this evolutionary trait impedes social-dependent tumor growth slowdown.

## Introduction

Social environment, psychological well-being and physiological functions are interconnected processes that potentially impinge on health and on disease outcomes. To investigate these interconnections, animal models provide appropriate strategies. For example, in mammals, stresses induced by social isolation or overcrowding have been associated with faster progression of type 2 diabetes^1^, cardiovascular or cerebrovascular disorders^2^ and metabolic like-syndrome^3^. Our previous work revealed that the growth of genetically-induced intestinal tumors in *Drosophila* depends on the social environment^4^. Tumorous flies exhibit increased social interactions, yet the underlying mechanisms linking the social context to tumor growth remain unexplained. This effect might be induced by an external signal, such as a pheromone-like product or the microbiota, which could directly affect host physiology to slowdown tumor growth. Alternatively, cognitive perception of the social context might generate a neurological relay modulating a tumor-suppressive physiological response.

Social behavior in the entomological world extends beyond eusocial insects^5,6^. Among others, studies with the *Drosophila melanogaster* model reveal that the fruitfly exhibits social learning^7^, aggregation behaviors^8^ and social-dependent decisions^9^. Reproduction-related behavioral changes have also been reported in fruitfly females, which after mating tend to aggregate on appropriate egg-laying sites in a pheromone-dependent signal, thereby increasing the expected rate of offspring survival^10–12^. Furthermore, in numerous animal species, mated females behave aggressively towards other females to acquire resources and protect their offspring^13,14^. This aggressive behavior also happens in mated *Drosophila* females, where it is induced by molecules transferred through the male ejaculate^15^. The most important ejaculate molecule is the sex-peptide that controls a number of post-mating changes, including high-rate egg laying, decreased receptivity to re-mating^16^, macronutrient usage^17^, increased midgut volume^18^, aggression towards other females^15^ and improved learning^19^. In addition, sperm by-products have been shown to affect female health and lifespan in several animal species, including *Drosophila melanogaster*^20,21^. These behavioral and physiological changes referred to as “male manipulation”, have likely been evolutionarily selected to promote male gene transmission^22^. In sum, the impact of male manipulation induces a sexual conflict between male and female gene transmission at the expense of female physiology^23^.

Our present study was initially designed to decipher how tumorous *Drosophila* individuals perceive their social environment to modulate tumor growth. To address this issue, we took advantage of the genetically-induced intestinal tumors model^24^ that we previously used to show that tumor growth is reduced when eight tumorous females were maintained in a homogenous group, compared to tumorous females maintained alone, either in isolation or in a heterogeneous group with seven non-tumorous control females^4^. Here, we show that this effect is independent of remnant pheromone-like products and of the microbiota. Transcriptome from the heads of isogenized tumorous females revealed significant changes in gene expression dependent on the social context, affecting various biological processes related to nervous system activity and mating behavior. This latter unexpected finding prompted an investigation into the social-dependent effect regarding reproductive status. Surprisingly, we found that the social effect on tumor growth occurs in virgin but not in mated females due to the influence of the sex-peptide. Given that reproduction may affect female health, in particular lifespan^20,25^, our study highlights another negative impact of female fertilization, which is the loss of the positive effect induced by social interactions to slowdown tumor growth.

## Results

### Social-dependent tumor suppression depends neither on remnant pheromone-like products nor on the microbiota

**Figure 1:**
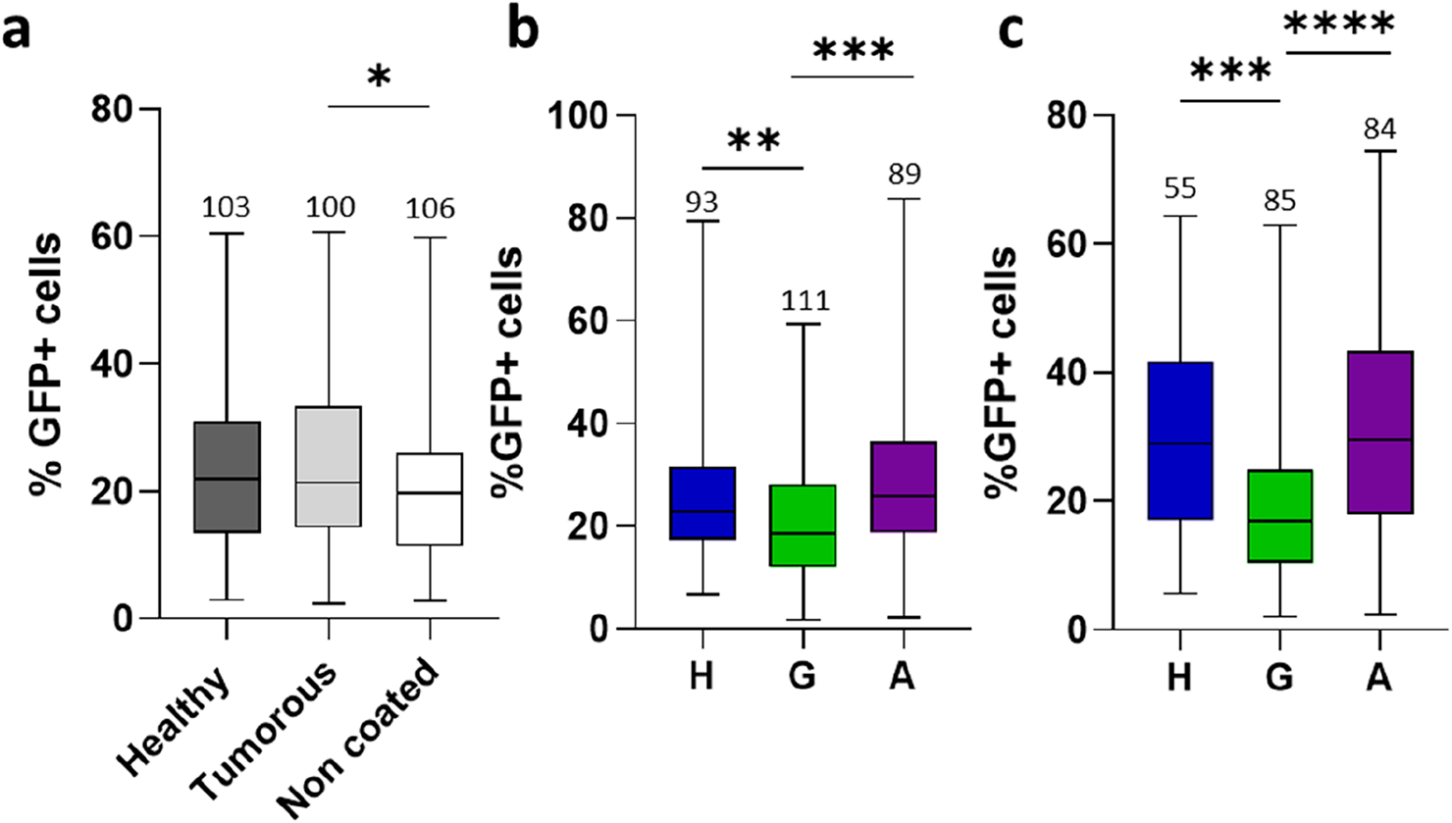
Social effect on tumor growth is independent of remnant pheromone-like products and of the microbiota. (**a**) Single tumorous flies were maintained for 24 days past heat-shock induction of the tumor in tubes either non-coated or previously coated by control or tumorous flies. (**b**,**c**) Axenic (**b**) and non-axenic (**c**) tumorous flies were maintained for 24 days in H-, A- or G-social conditions: heterogeneous, alone or homogenous group, respectively. In all experiments, flies were transferred to new vials twice a week. Given that tumors are GFP-labeled, their size was monitored by flow cytometry. Boxplots represent the distribution of percentages of GFP-positive cells reported to the total number of cells from dissected midguts plus the Malpighian tubules; boxes correspond to the quartiles of the distribution (25%, median and 75%), wiskers extend to the extreme values. The numbers of gut analyzed individually for each condition are indicated at the top of each box. Percentages of GFP-positive cells for each replicates are listed in S4 Table.

To determine whether the social-induced effect on tumor growth^4^ depends on environmental signals, we first challenged remnant pheromone-like products. The fly lines were isogenized prior to the experiments reported in this study that spanned a 2-year period. Previous studies reported that certain molecules, including cuticular hydrocarbons/pheromones and autotoxins^26,27^, can persist on tube walls after fly removal. To assess the impact of potential pheromone-like products, eight virgin females, either control or tumorous, were maintained in different vials for one day to serve as “coating flies”. After discarding these coating-flies, single newly induced tumorous flies were placed in these vials, while additional tumorous flies were placed alone in non-coated vials. These tumorous flies were maintained alone during 24 days past tumor induction, with vials changed twice a week to ensure exposure to coated or non-coated environments. Then, midgut were dissected, enzymatically digested to single cells and the percentage of GFP-labeled tumor cells was measured by FACS, as previously described^4^. In this setting, no significant differences in tumor size were observed between flies maintained in either control- or tumorous-coated vials, whereas tumor size was significantly reduced in flies maintained in non-coated vials (Fig. 1a). The observed decrease in tumor growth for “non-coated flies” could possibly result from reduced nutrient access. As reported in numerous insect species, *Drosophila* has been shown to regurgitate digestive enzymes from the salivary glands^28–30^. Thus, flies used for coating can pre-digest the feeding media. Then, nutrient assimilation should be favored for the single tumorous flies reared in coated vials and potentially promoting tumor growth. Conversely, single tumorous flies reared in non-coated vials may not benefit from pre-digested food, leading to delayed nutrient assimilation and subsequent tumor growth. Nonetheless, these results exclude a potential role of remnant molecules specific of tumorous but not control flies, which might restrain tumor growth in tumorous flies raised in homogenous group.

Next, to explore whether differences in the microbiota may be responsible for the social-induced slowdown of tumor growth, experiments were conducted using axenic flies. Axenic control and tumorous flies were generated as described^31^ and raised in three social conditions: alone (A) or in group of eight flies, either homogenous (G: eight tumorous flies) or heterogeneous (H: one tumorous with seven control flies)^4^. The drastic effect on bacterial colony formation confirmed the axenic conditions (Supplementary Fig. 1). Importantly, tumor growth of G-flies was significantly reduced compared to H- and A-flies (Fig. 1b), and this effect mirrored the one observed for non-axenic flies (Fig. 1c). Collectively, these findings indicate that the slowdown of tumor growth is independent of the microbiota and of remnant pheromone-like products.

### Social context impinges on nervous system-related functions

**Figure 2:**
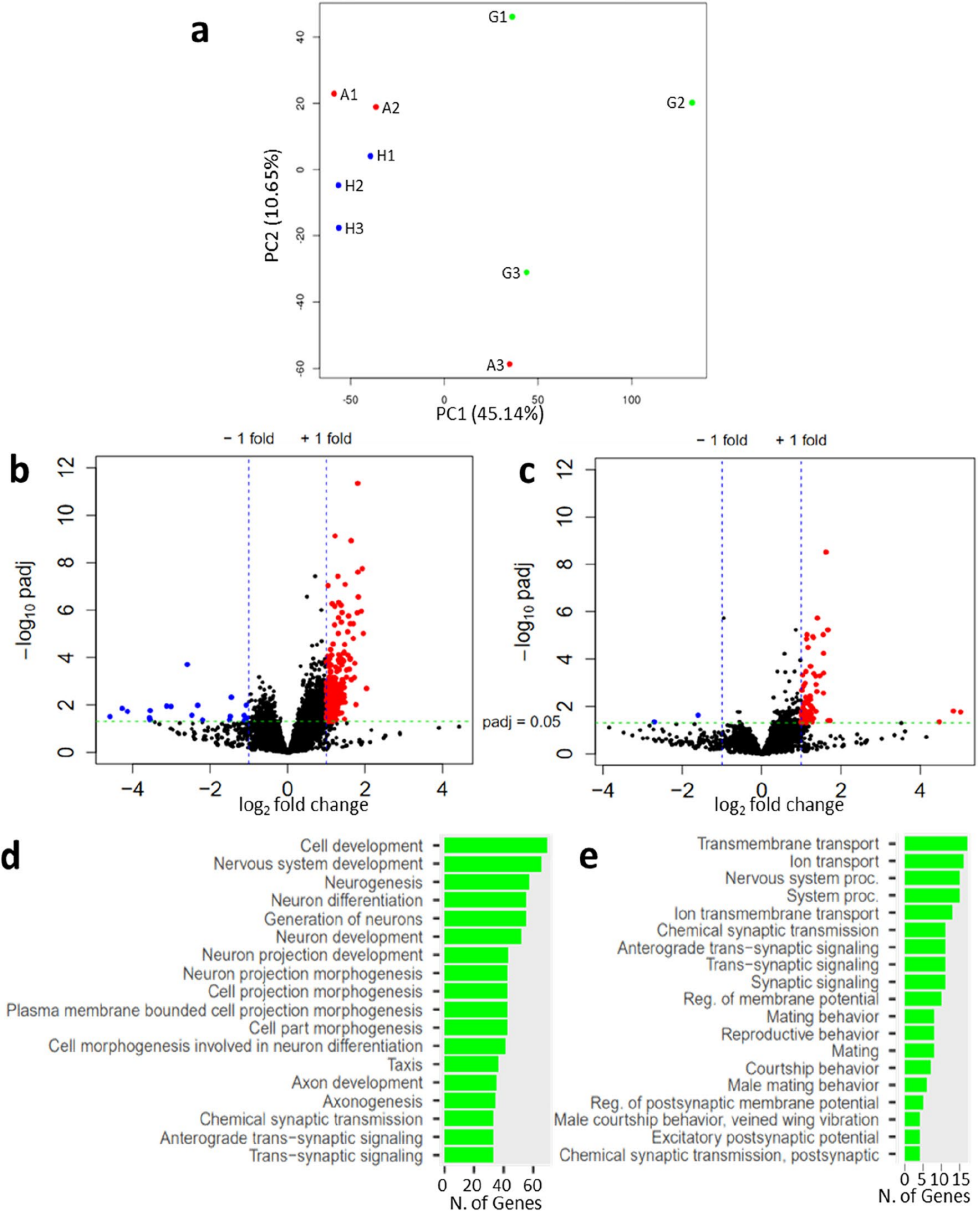
Transcriptomes (RNAseq) from the heads of G- H- and A-tumorous flies. (**a**) Principal components analysis comparing G-, H- and A-replicates, corresponding to heterogeneous, alone or homogenous groups, respectively. (**b**,**c**) Volcano plots showing significantly up-(red) and down-(blue) regulated genes when comparing H- to G-conditions (**b**) and A- to G-conditions (**c**). (**d**,**e**) Biological processes identified when analyzing through ShinyGO the genes differentially expressed in H- (**d**) and A-flies (**e**) compared to G-flies.

To investigate whether a brain integration of the social context occurs in tumorous flies, we performed transcriptome analysis from the heads of 100 flies for each replicate (three replicates per condition). We selected the 10-day time point based on our previous study^4^, where we observed a noticeable increase in tumor growth between days 7 and 14 post tumor induction. Additionally, we hypothesized that the social impact might not manifest immediately, requiring a certain time lapse to significantly influence gene expression. Finally, we assumed that the social-dependent effect operates throughout the entire process of tumor growth rather than at a specific stage. Thus, we estimated that he social impact on gene expression and subsequently on tumor growth was likely established 10 days post heat shock. Given the isogenic nature of the fly lines, differences between conditions result solely from the social context in which flies are distributed after tumor induction. Principal component analysis revealed distinct segregation of the three replicates of H(heterogeneous)-flies from the three replicates of G(group)-flies (Fig. 2a), whereas A(alone)-replicates segregate in between the G- and H-replicates, although closer to the latter (Fig. 2a). Differentially expressed genes were selected based on a P_adjusted_ < 0.05 and on a Log2foldchange > ǀ1ǀ. In this way, we found that the expression of 251 genes was significantly different in H- compared to G-replicates, with 231 genes up-regulated and 20 genes down-regulated (Table S1). Comparisons between A- and G-replicates revealed significant changes in the expression of 78 genes, with 76 up-regulated and 2 down-regulated (Table S2). Further investigations will be needed to understand why the vast majority of gene expression changes are associated with up-regulation. In contrast, no significant differences in gene expression were observed when comparing H- to A-flies (Table S3). Volcano plots visually represent these significant differences in gene expression when comparing H- or A- to G-flies (Fig. 2b,c), while no differences are evident when comparing H- to A-flies (Supplementary Fig. 2). Gene ontology analysis of the genes differentially expressed in H- versus G-flies, using ShinyGO^32^, highlights biological processes related to neuronal activities (Fig. 2d). Similar analysis of the genes differentially expressed in A- versus G-flies highlights biological processes related to neuronal activities and to mating behavior (Fig. 2e).

In sum, these analyses reveal significant changes in the expression of genes involved in nervous system-related activities when comparing G-flies to either H- or A-flies, but not when comparing A- to H-flies. These differences indicate that tumorous flies perceive a cognitive signal from their tumorous conspecifics that might relay the social context to the growth control of their intestinal tumors.

### Fertilization impedes social-induced tumor slowdown

**Figure 3:**
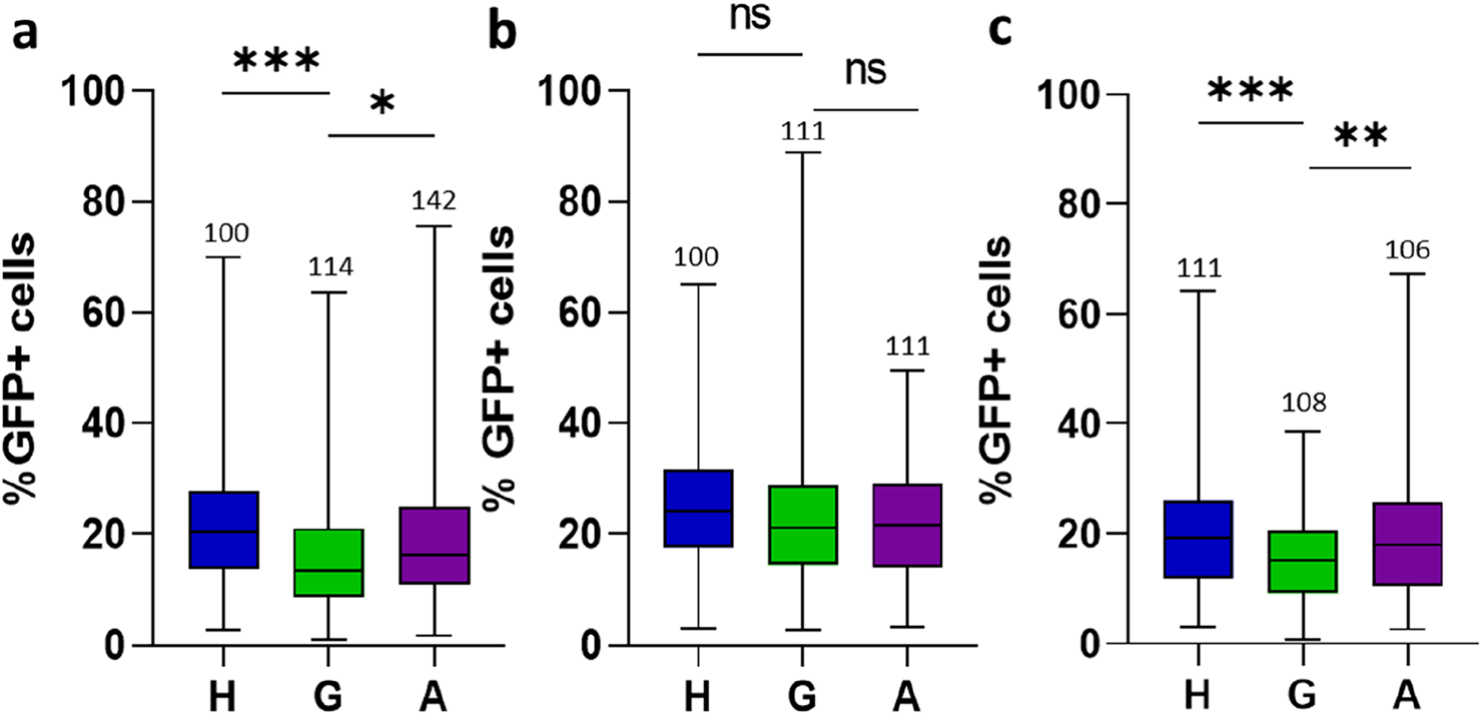
Social effect on tumor growth is lost in fertilized females. (**a**–**c**) Virgin females (**a**) and females fertilized by either wild-type (**b**) or SP0 (**c**) males were maintained for 24 days past heat-shock induction of the tumor in H-, A- or G-social conditions. Measurements of tumor growth and their representations are similar to those of Fig. 1. Percentages of GFP-positive cells for each replicates are listed in S5 Table.

The RNAseq analysis highlights biological processes related to neuronal activities but also to reproductive-dependent behaviors. As with the experiments reported above, our previous study was performed with virgin *Drosophila* females^4^. Importantly, numerous changes have been described after female fertilization, including modification in (i) behavior^15^, (ii) gene expression related to the nervous system^33–35^, (iii) the immune response^21,36^ and (iv) intestinal physiology^37^. Therefore, we wondered to what extent the social effect on intestinal tumor growth would operate in fertilized *Drosophila* females. To address this issue, newly emerged females were paired with wild-type males for approximately 2 days. After heat-shock-dependent tumor induction, fertilized flies were subjected to the A-, H- and G-social conditions, with the distinction that the seven healthy flies for the H-condition were fertilized. Remarkably, tumor size was not significantly different in any of the social condition (Compare Fig. 3a–b). Givent that numerous changes induced by fertilization, including behavior and physiology, are due to the sex-peptide transmitted to the female through the male ejaculate^15,16,18,22^, we repeated the experiment with tumorous females fertilized by sex-peptide deficient (SP0) males. In this setting, the reduced tumor growth observed in G-flies was restored (Fig. 3c), indicating that the lack of effect in fertilized females is directly linked to the sex-peptide transferred via the male ejaculate. These findings imply that, in addition to its plethoric effects^22^, the sex-peptide impinges on the social-induced slowdown of tumor growth.

### Behavioral changes in fertilized tumorous females

**Figure 4:**
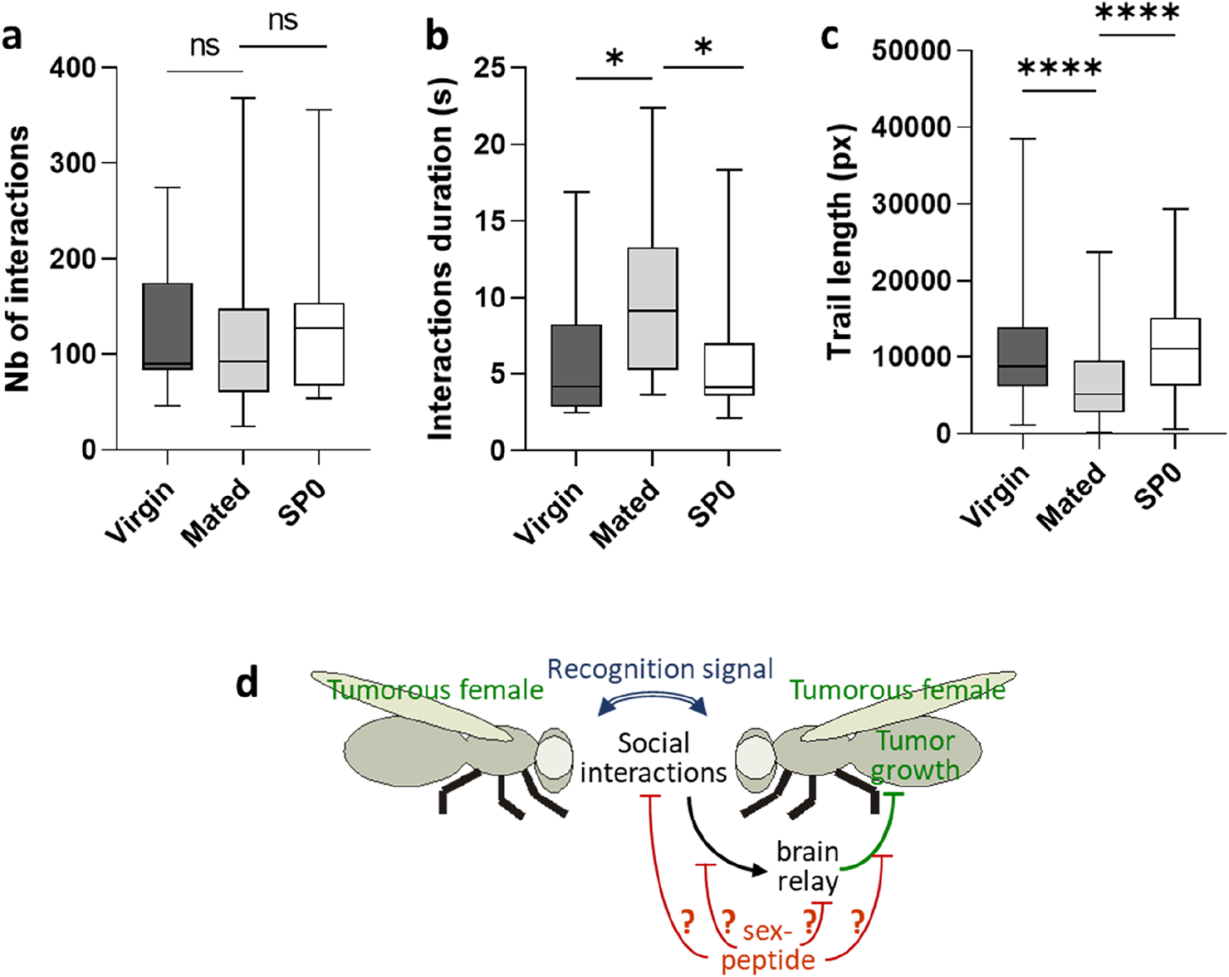
Behavior is modified in tumorous fertilized females. (**a**) Numbers of contacts an individual has with other flies in the arena. (**b**) Durations of the contacts an individual has with other flies in the arena. (**c**) Total locomotion trails. Virgin or females fertilized by either wild-type (mated) or SP0 males, were tracked for 1 h with synchronized firewire cameras; 16 replicates were analyzed for each condition. Boxplot representations are similar to those of Fig. 1. (**d**) Putative model for tumor growth slowdown in response to social interactions: tumorous females recognize each other through an as-yet-unknown signal (blue), prompting social interactions, which in turn induce changes in the expression of neurological related genes (black). These gene-expression changes might mediate a relay to trigger a physiological response slowing down tumor growth (green). The sex-peptide might affect the social interaction, the neurological relay or the physiological response (red).

We previously reported distinct social interactions in G-tumorous flies compared to G-control flies^4^. In addition, sex-peptide-dependent behavioral changes have been previously reported^15,16,19^. Therefore, we wonder whether behavioral changes also occurs in fertilized tumoral females. To address this issue, we monitored social interactions between G-tumorous flies, either virgin or fertilized by wild-type or SP0 males. In contrast to our previous study^4^, where homogenous groups of eight flies were established from eight different groups of flies 21 days past tumor induction, social monitoring in this experiment was performed with flies maintained in the same social group for 4–6 days past tumor induction. These experiments were performed early after tumor induction, because the sex-peptide effect decreases one week after fertilization^22^. No differences were observed in the number of interactions between virgin and fertilized flies with either wild-type or SP0 males (Fig. 4a). Conversely, females fertilized by wild-type males exhibited an extended duration of their interactions (Fig. 4b) alongside a decrease in their total locomotion trail (Fig. 4c) compared to virgin and SP0-fertilized females (Fig. 4c). Congruent with what has been described in wild populations^11,16,22^, these results confirm that tumorous flies undergo post-mating behavioral changes in response to the sex-peptide. In summary, these findings reveals that in response to the sex-peptide, fertilized females might either perceive their social environment differently or have lost the physiological control of tumor growth depending on the social context.

## Discussion

In this study, we demonstrate that the social-dependent effect on tumor growth in genetically identical tumorous flies is independent of remnant pheromone-like products and of the microbiota. Conversely, we provide evidence that the social environment in which tumorous flies are raised can modify the expression of genes associated with neurological functions. Notably, significant gene expression changes are observed when comparing G (homogenous)-flies to A (alone)- or H (heterogeneous group)-flies, but not when comparing A- to H-flies. As suggested in our previous study^4^, this observation supports the notion that tumorous flies in heterogeneous groups perceive their social environment similarly to isolated flies. Therefore, it is tempting to speculate that the slowdown of tumor growth induced by the social context potentially depends on a neurological relay, although cognitive perception of the social environment needs formal demonstration through additional studies in *Drosophila*. In our previous work^4^, we found that, in dual choice tests, young tumorous virgin females tend to prefer the environment of their siblings. The experimental procedure ruled out a physical or a visual signal as responsible for this social behavior. Taken together, these findings suggest that tumorous flies can recognize their tumorous siblings, promoting social interactions that, in turn, lead to changes in the expression of genes related to neurological functions (Fig. 4d). Nevertheless, the signal responsible for tumorous fly recognition may differ from the one induced by social interactions, which triggers a neurological relay, subsequently promoting tumor growth slowdown. Therefore, future investigations should be designed to determine whether these social-dependent changes in gene expression are induced by a non-persistent olfactory signal, a visual signal, a sound signal or a direct physical contact.

Unexpectedly, transcriptome analysis revealed changes in the expression of genes involved in mating behavior. In addition, the social-dependent tumor growth slowdown is absent in mated females, because of the sex-peptide. Peptides transferred via the male ejaculate induce numerous behavioral changes^11,16,22^. For instance, mated females tend to aggregate on appropriate egg-laying sites in response to a pheromone-dependent signal, thereby increasing the expected rate of offspring survival^10–12^. However, as described in numerous animal species^13,14^, mated *Drosophila* females may behave aggressively towards other females to acquire resources and protect their offspring^15^. Consistently, we observed that tumorous flies exhibit post-mating behavioral changes in response to the sex-peptide. However, in contrast to our previous study suggesting that increased social interactions induce a tumor suppressive effect^4^, our present findings indicate that this correlation should be approached cautiously. In our previous study, all the flies exhibited the same total locomotion trail, while in the present one, mated flies show a decrease in locomotion trail concurrent with an extended contact duration. Therefore, the extended contact duration in mated females might result from their gregarious behavior, highlighting that an increase in social interaction is not necessarily correlated with a tumor suppressive effect. Further experiments should be designed to compare the aggressiveness of the social contacts between mated and virgin females and to determine whether the same social-dependent changes in gene expression occur in mated and virgin females.

The sex-peptide dependent behavioral and physiological changes, referred to as “male manipulation”, have likely been evolutionarily selected to promote male gene transmission^22^. This phenomenon induces a sexual conflict between male and female gene transmission at the expense of female physiology^23^. Here, we show that male manipulation via the sex-peptide operates at the expense of the social-induced slowdown of tumor growth. Whether fertilized females perceive their social environment differently or have lost the social-induced physiological response controlling tumor growth remains an open question. Due to pleiotropic effects^11,16,22^, the sex-peptide might impinge on the social-dependent tumor growth-suppression through multiple inputs, including changes in social interactions, cognitive perception, gene expression, and antitumoral physiological response (Fig. 4d). In summary, our study highlights a physiological axis linking behavior, cognition, reproduction and antitumoral response. Thus, we postulate that male manipulation, likely selected to favor male gene transmission, results in an evolutionary conflict between reproductive investment and tumor growth fighting in *Drosophila* females.

## Methods

### Genetics

To generate tumors, MARCM clones were randomly induced by flipase-dependent recombination in the offspring of *yw,HS-flp*;*esg-gal4*,*UAS-GFP*;*FRT82B*,*Tub-Gal80* females mated to *yw,HS-flp*;*UAS-Ras*^*V12*^;*FRT82B*,*Apc2*^*N175K*^*,Apc*^*Q8*^ males, as previously described^4,24^. Both lines were isogenized prior to all the experiments reported in the study, which spanned a two-year period. Isogenized lines were established from unique X, II and III chromosomes by crossing heterozygote males to females bearing balancer chromosomes. Flipase expression was heat-shock induced in 2–3 days old females. Control *w*^*1118*^ flies were used as healthy females in H-groups and as wild-type males for mating. SP0 males were the F1 progeny from *SP0/TM3,Sb* females crossed to Δ*130/TM3,Sb* males^19^. Immediately after flipase-induced recombination, females were split in homogenous group of eight tumorous flies (G) or as single flies, either alone (A) or in heterogenous group with seven healthy flies. Fertilized females were mated with males for 2–3 days, prior to the heat shock and males were removed. Flies were kept in a 25 ℃ incubator and changed twice a week into new vials containing our standard media^38^.

### Flow cytometry

24 days past flipase induced recombination, midguts and Malpighian tubules—both contain tumors—from individual flies were dissected and digested to single cells by sequential treatment with Collagenase and trypsin (Sigma–Aldrich) at 27 ℃, as previously described^4^. Sample containing the intestinal cells of single flies were treated on a Flowlogic 7.2 (Miltenyi–Biotech/lnivai) cytometer and analyzed with CytExpert 2.4. Results are presented as mean percentages of tumorous cells, which are labelled by GFP, reported to the total cell number. We noticed that effects were visible with 50 replicates (flies), but for the robustness of our statistical values, about 100 flies were analyzed for each condition in all the experiments; so that each experiment lasted about three months to get enough flies.

### Axenic flies

Parental flies were let to lay eggs for 12 h on grape juice plates as described^39^. Eggs were collected and sequentially incubated 2 min in 2% bleach, 2 min in 70% ethanol and 2 min in sterile water. Next eggs were transferred in UV sterilized vials containing autoclaved standard media supplemented with antibiotics (50 μg/mL ampicillin, 50 μg/mL kanamycin, 15 μg/mL erythromycin, 50 μg/mL tetracyclin) as described^31^. Emerging flies were manipulated in alcohol disinfected area and the following experiments were done with the above-described vials. To test the axenic efficacy (Supplementary Fig. 1), four adult females were crushed in 500 μL Browth. 100 μL of extract were sequentially diluted and plated on standard agar plates and Colony forming unit (Cfu) were calculated as the number of colonies per mL of extract (Cfu standard: 1.9 × 10^6^/mL; Cfu axenic 4 × 10^3^/mL).

### Transcriptome analyses (RNAseq)

For each replicate, groups of 100 tumorous flies were beheaded on dry ice and the heads were store at − 80 ℃. These flies were selected 10 days post tumor induction, as we assumed that the social-induced effect occur along the entire process of tumor growth. RNA was extracted using Trizol–chloroform and libraries for Illumina sequencing were prepared using the Illumina TruSeq total RNA stranded kit^38^ and sequenced using a NextSeq 550 instrument. DNA reads were converted into raw data (FASTQ files) and preprocessed to remove adapters, low-quality sequences and artefacts. Cleaned sequences were aligned to the reference genome “dmel-all-chromosome-r6.42.fasta” using HISAT2. Differential analyses between replicates were performed using the DESeq2 tool to identify genes whose expression significantly differs between experimental conditions. Data were finally analyzed using R Studio^40^ and Gene Ontology (GO) was performed using using ShinyGO^32^.

### Behavior

Tumors were induced 2–3 days past adult eclosion in females either virgins or mated with SP0 or wild-type males. Immediately after heat-shock males were removed and females split in groups of eight flies of the same physiological status. 4–6 days later, these groups of flies were introduced into a semi-opaque white polyoxymethylene (Delrin) arena (diameter 100 mm; height 5 mm), covered with a transparent Plexiglas. We simultaneously tracked four groups of eight flies over 1 h. To prevent circadian dependent bias, experiments were performed in the afternoon between 1 and 4pm. The tracking apparatus consisted of four synchronized firewire cameras (Guppy pro, Allied vision technologies), each filming one arena that was backlit by a 150 × 150 mm IR backlight (R&D vision). We used Vision software to analyze spatial data (open-source C-trax 0.3.7)^41^ allowing collection of 10 frames per second. Tracking corrections were made post C-trax analysis with fixerrors Matlab toolbox (Ctrax-allmatlab version 0.2.11) using Matlab software 7.11.0 to generate the spatial coordinates of each fly. Total trail length, number of contacts between flies (a contact was defined as the distance between two individuals was less than one leg length for at least 1 s) and the duration of each contact were calculated with an algorithm written in R. Unpaired t-test analyzes were performed using GrahPad Prism. Significance was coded in the following way based on the p-values: ****p < 0.0001; ***0.0001 < p < 0.001; **0.001 < p < 0.01; *0.01 < p < 0.05.

### Supplementary Information

Supplementary Information.

Supplementary Table S1.

Supplementary Table S2.

Supplementary Table S3.

Supplementary Table S4.

Supplementary Table S5.

## Data Availability

Most data generated during and/or analyzed during the current study are included in this published article and its supplementary information files, including the R-script used for processing the raw data tables supporting the Fig. 4; corresponding raw data tables and videos are available upon request to the corresponding author.
