# Supplementary material for: Male manipulation impinges on social-dependent tumor suppression in Drosophila melanogaster females
Source: Sci Rep. 2024 Mar 17;14:6411. doi: 10.1038/s41598-024-57003-3 (PMC10944827; doi:10.1038/s41598-024-57003-3)
Supplement: Supplementary file 1 — Supplementary Information. [file 41598_2024_57003_MOESM1_ESM.pdf]

Male manipulation impinges on social-dependent tumor suppression in *Drosophila melanogaster* females

Perla Akiki, Pierre Delamotte, Mickael Poidevin, Erwin L. van Dijk, Apolline J. R. Petit, Arnaud Le Rouzic, Frederic Mery, Frederic Marion-Poll, Jacques Montagne

Supplementary Table legends and Supplementary Figures:

Supplementary Table 1: Differential gene expression when comparing H(Heterogeneous)- to G(Group)-conditions. Gene sorting follows the increase in P-value. Genes whose expression if significantly up(red) and down(blue)-regulated are underlined.

Supplementary Table 2: Differential gene expression when comparing A(Alone)- to G(Group)-conditions. Gene sorting follows the increase in P-value. Genes whose expression if significantly up(red) and down(blue)-regulated are underlined.

Supplementary Table 3: Differential gene expression when comparing H(Heterogeneous)- to A(Alone)-conditions. Gene sorting follows the increase in P-value. Note the absence of gene differentially expressed based on a Pvalue <0,05 and on a Log2foldchange >│1│.

Supplementary Table 4: Data used to generate Fig. 1. Each value represent the percentage of GFP+ (tumorous) cells from dissected guts individually analyzed by flow cytometry after enzymatic digestion of the extra-cellular matrix.

Supplementary Table 5: Data used to generate Fig. 3. Legend similar to supplementary Table 4.

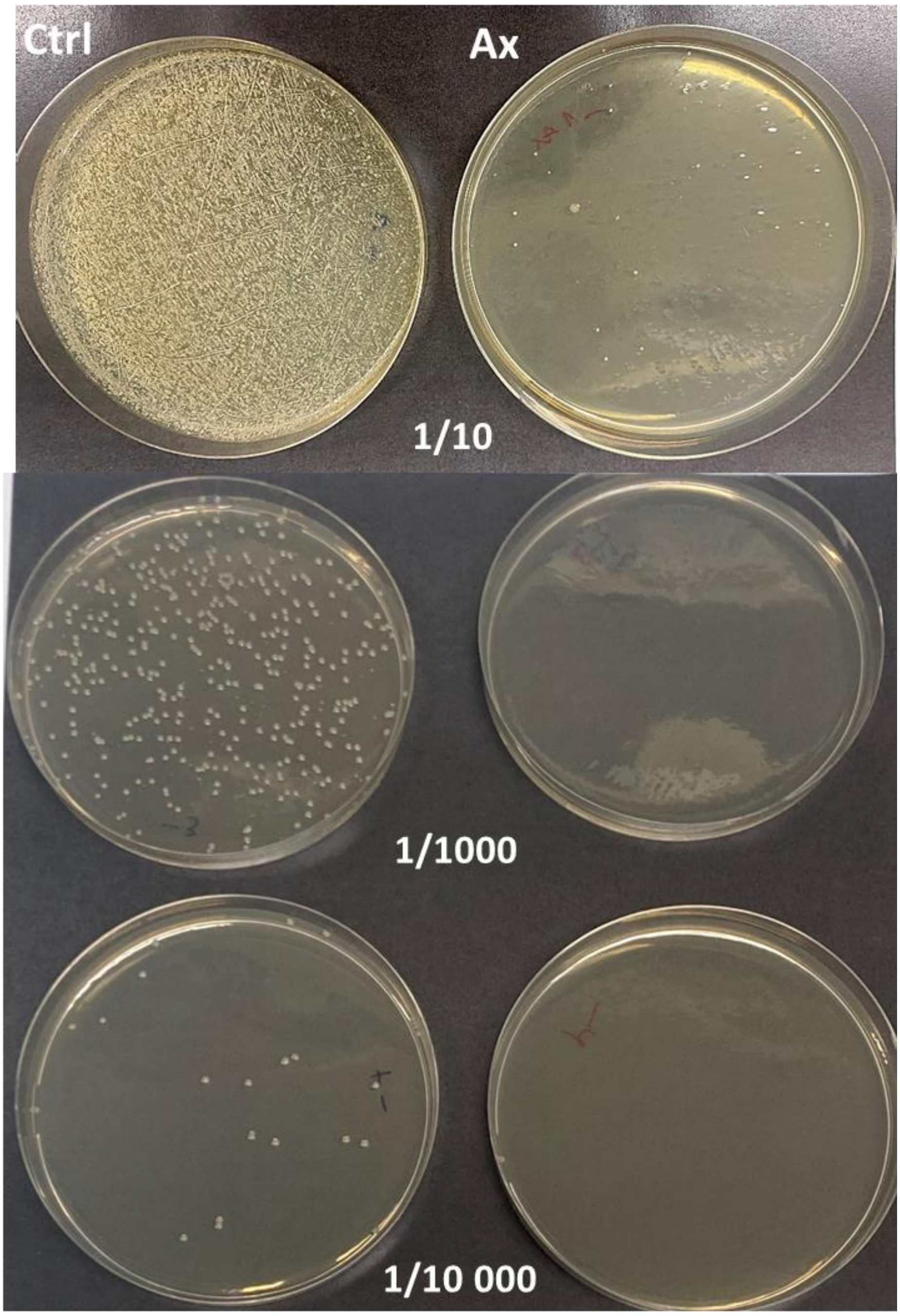

Supplementary Fig. 1: Tests for axenic efficacy. 4 adult females either control (left) or axenic (right) were crushed in 500 µl PBS. 100 µl of diluted solutions were plated on standard agar plates at the indicated dilutions.

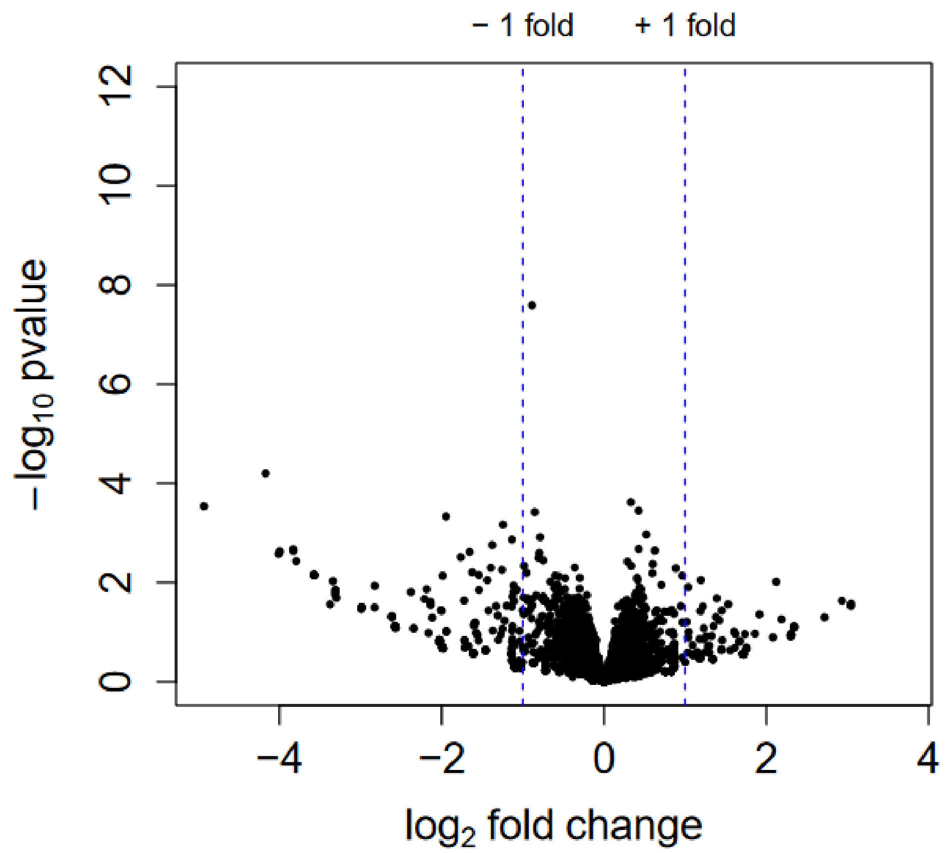

Supplementary Fig. 2: Volcano plots comparing H(Heterogeneous)- to A(Alone)-conditions. Note that based on a Pvalue <0,05 and on a Log2foldchange >│1│, none of the difference in gene expression is significant.

Supplementary code for analyzing social interactions

```
## Social Interactions - Nov. 2021  ##

## Packages to load once ##
rm(list=ls())
install.packages("dplyr")
install.packages("tidyr")
install.packages("stringr")
install.packages("ggplot2")

############################

## Settings ##
library("dplyr")
library("tidyr")
library("stringr")
library("ggplot2")
setwd("Write_working_directory_here")

name_file_input <- "name_experiment.csv" ## PUT NAME OF THE FILE HERE
<<<<<<<<<<<<<<<<<<<<<<<<<<<<<<<<<<<<<<<<<<<<<<<
d_interaction <- 2.5 ## DEFINE THE MINIMUM DISTANCE OF INTERACTION HERE
<<<<<<<<<<<<<<<<<<<<<<<<<<<<<<<<<<<<<
t_interaction <- 9 # DEFINE THE MINIMUM CONTACT DURATION HERE
<<<<<<<<<<<<<<<<<<<<<<<<<<<<<<<<<<<<<<<<<<<<<
name_file_output_raw <- "File name"
name_file_output_summary <- "File name_summary"


## Data loading##
tab2 <- read.csv2(name_file_input, header=F, sep=";", dec=".")

## Defining new table with headers ##
names_tab <- c("ID", "X", "Y", "Size_width", "Size_height", "Angle")
m <- length(tab2[1,])/6 # 6 is the number of measurements per fly ; m the
number of flies
header <- rep(names_tab, times=(m))
colnames(tab2) <- header
tab2 <- as.data.frame(tab2)
tab3 <- NULL
for (j in 0:(m-1)){
  tab3 <- rbind(tab3, tab2[,(6*j+1):(6*j+6)])
}

## Adding time ##
Time_int <- c(1:length(tab2[,1]))
Time <- rep(Time_int, times=m)
tab3 <- cbind(tab3, Time)
tab3 <- as.data.frame(tab3)

## Ordering data according to time points ##
tab3 <- tab3[order(tab3$Time),]

## Quality control ##
flies <- as.numeric(as.character(names(table(tab3$ID))))
ggplot(tab3, aes(x=ID))+
```

```
  geom_histogram(binwidth=0.5)+
  scale_x_continuous(breaks=flies)+
  stat_bin(aes(y=..count.., label=ifelse(..count..==0,"",..count..)),
geom="text", vjust=-.5)+
  labs(title="Number of measurments per fly", x = "Fly ID", y = "Counts")

## Analyzing ##
## Creating the new table with interactions per fly ##
comb <- c()
n_comb <- (factorial(m))/(2*(factorial(m-2)))
for (k in 1:(m-1)){
  for (l in (k+1):(m)){
    comb <- c(comb, paste0("Comb",k,"-",l))
  }
}
comb <- c(comb, "Time")
tab4 <- as.data.frame(matrix(ncol=(n_comb+1), nrow=0))

## Filling the new table with distances to be analyzed ##
# Indexes for each series of flies are calculated in a loop to directly
pick up the indexes. Example: with 7 flies, m=7.
ind_min <- c()
ind_max <- c()
for (o in 0:(max(Time_int)-1)){
  ind_min <- c(ind_min, (m*o+1))
  ind_max <- c(ind_max, (m*o+m))
}

# Calculating measures thanks to the correct indexes previously calculated
and deleting those harboring "-1"
for (p in 1:max(Time_int)){
  distances <- c()
  for (q in (ind_min[p]):(ind_max[p]-1)){
    for (r in (q+1):(ind_max[p])){
      if (tab3$X[r] > 0 && tab3$X[q] > 0 && tab3$Y[r] > 0 && tab3$Y[q] >
0){
        distances <- c(distances, sqrt(((tab3$X[r]-
tab3$X[q])^2)+((tab3$Y[r]-tab3$Y[q])^2)))
      }else {
        distances <- c(distances, 0)
      }
    }
  }
  distances <- c(distances, p)
  tab4 <- rbind(tab4,distances)
}
colnames(tab4) <- comb

## Calculating Interactions ##
## if interaction, return 1 ; otherwise return 0 ##
tab5 <- tab4
for (s in 1:nrow(tab4)){
  for (t in 1:((ncol(tab4))-1)){
    if (tab4[s,t] <= d_interaction){
      tab5[s,t] <- 1
    }else{
```

```
      tab5[s,t] <- 0
    }
  }
}

## Calculating number of interactions and contact duration ##
## Concatenate the number of consecutive 1 and give the length of each
interaction ("interactions") vector containing the number of interactions
("indexes")
interactions <- c()
indexes <- c()
all_comb <- c()
for (u in 1:((ncol(tab5))-1)){
  count <- 0
  for (v in 1:nrow(tab5)){
    if (tab5[v,u] == 0 && count <= t_interaction){
      count <- 0
    } else if (tab5[v,u] == 0 && count > t_interaction){
      interactions <- c(interactions, count)
      count <- 0
    } else {
      count <- count+1
    }
  }
  if (count > t_interaction && tab5[v,u] == 1){
    interactions <- c(interactions, count)
  }else if(length(interactions) == 0){
    interactions <- c(0)
  } else {
    interactions <- c(interactions)
  }
  indexes <- c(indexes, length(interactions)) #if interactions is not
reinitialized, keep indexes to know the number of interaction per
combination of flies
  all_comb <- c(all_comb, interactions)
  interactions <- c()
}

## Creating the final table with individual interactions and calculated
interactions for each fly ##
# Adding the previous values for each combination
tab6 <- as.data.frame(matrix(NA, nrow=length(all_comb), ncol=ncol(tab5)-
1+m))
for (z in 1:length(indexes)){
  tab6[1:(indexes[z]),z] <- all_comb[1:indexes[z]]
  all_comb <- all_comb[-(1:indexes[z])]
}
# Preparing the labels for each fly (couples of interactions will be
associated for each fly)
fly <- c()
for (w in 1:m){
  fly <- c(fly, paste0("Fly ",w))
}
labels_tab6 <- c(colnames(tab5[1:(ncol(tab5)-1)]), fly)
colnames(tab6) <- labels_tab6
```

```
# Adding the interactions for each fly
detect_fly <- c()
detect_fly2 <- c()
cumul <- c()
index_fly <- NULL
for (a in 1:m){
  detect_fly <- str_detect(colnames(tab6), str_c(a)) &
str_detect(colnames(tab6), str_c("Comb"))
  detect_fly2 <- as.numeric(as.character(which(detect_fly == TRUE)))
  index_fly <- as.numeric(as.character(which(str_detect(colnames(tab6),
str_c(a)) & str_detect(colnames(tab6), str_c("Fly")) == TRUE)))
  for (b in 1:(length(detect_fly2))){
    cumul <- c(cumul, as.numeric(na.omit(tab6[,(detect_fly2[b])])))
  }
  tab6[,index_fly] <- c(cumul, rep(NA, (nrow(tab6)-length(cumul))))
  cumul <- c()
}
tab6[tab6 == 0] <- NA
# Final analysis - Summary
tab7 <- tab6[,as.numeric(as.character(which(str_detect(colnames(tab6),
str_c("Fly")) == TRUE)))]
tab7 <- tab7/10
n_interactions <- c()
new_names <- c()
all_interactions <- c()
means_times <- c()
for (c in 1:ncol(tab7)){
  n_interactions <- length(as.numeric(na.omit(tab7[,c])))
  all_interactions <- c(all_interactions, n_interactions)
  means_times <- c(means_times, mean(tab7[,c], na.rm=T))
  new_names <- c(new_names, paste0("Fly ",c," ; ",n_interactions, "
interaction(s)"))
}
colnames(tab7) <- new_names
tab7[] <- lapply(tab7, function(x) c(x[!is.na(x)], x[is.na(x)]))
# To write additional calculations within tab7
summ <- c(rep(NA,nrow(tab7)))
names(summ) <- "summary"
summ[1] <- c("Mean nb of interactions")
summ[2] <- c(mean(all_interactions))
summ[3] <- c("-")
summ[4] <- c("Mean of interactions duration")
summ[5] <- c(mean(na.omit(means_times)))
summ[6] <- c("-")
tab7 <- cbind(tab7, summ)
#-----------------------------------------#
summary_tab <- summary(tab7[,-(8:9)])
write.table(tab7, file= name_file_output_raw, sep ="\t", row.names=T)
write.table(summary_tab, file= name_file_output_summary, sep ="\t",
row.names=F)
```
